# Exploring exclusive breastfeeding readiness: experiences of first-time mothers in Lusaka, Zambia

**DOI:** 10.1080/17482631.2025.2463159

**Published:** 2025-02-09

**Authors:** Francis Sichimba, Kalunga Cindy Nakazwe, Lena Halawi, Atika Khalaf

**Affiliations:** a Department of Psychology, University of Zambia, Lusaka, Zambia; b Faculty of Health Sciences, Kristianstad University, Kristianstad, Sweden; c Department of Nursing, Fatima College of Health Sciences, Ajman

**Keywords:** Care, exclusive breastfeeding, experiences, first-time mothers, readiness

## Abstract

**Background:**

The World Health Organization recommends exclusive breastfeeding for the first six months of a baby’s life. While many studies have provided insight into exclusive breastfeeding in Zambia, few have looked at the experiences of first-time mothers and exclusive breastfeeding readiness.

**Objective:**

To explore exclusive breastfeeding readiness and experiences of first-time mothers in Zambia.

**Methods:**

A qualitative descriptive study employing a phenomenological design was conducted with 17 mothers. Data was collected using a semi-structured face-to-face interview guide. Thematic analysis was used to analyse the interview transcripts.

**Results:**

The analysis resulted in an overarching theme: *Breastfeeding Readiness—A Multifaceted Approach* with four categories: Exclusive Breastfeeding Readiness and Motivation, Support Networks in the Breastfeeding Journey, Navigating the Exclusive Breastfeeding Journey, and Perceived Resources to Support Exclusive Breastfeeding Readiness.

**Conclusions:**

Based on the findings, it is evident that although first-time mothers have knowledge on exclusive breastfeeding, mentally and practically, they are not ready to do so successfully. The study recommends improved prenatal and postnatal care provided to new mothers, with a focus on mental health readiness, breastfeeding instruction, and skills development.

## Introduction

### Background to area of study and problem description

Breastfeeding remains a public health issue on account of its importance in infant care and management (Pérez-Escamilla et al., 2023). The 2030 Agenda for Sustainable Development under goal number three has earmarked breastfeeding as one of the United Nations’ goals (United Nations, 2016). The World Health Organization (WHO) recommends sustained exclusive breastfeeding, referring to both feeding at the breast as well as expressed milk feeding, for the first six months of life to provide the infant with natural nutrition to promote infant development (World Health Organization, 2024a). WHO further recommends that breastfeeding should continue for up to two years or longer and that nutritious complementary foods can be introduced at the age of six months (World Health Organization, 2024b). Despite many countries and organizations such as the United Nations Children’s Fund (UNICEF) championing exclusive breastfeeding, statistics show that exclusive breastfeeding rates among infants under the age of six months are at around 48.0% in most countries (UNICEF, 2023). Economically, low rates of exclusive breastfeeding are associated with yearly economic losses of between 37 and 70% of the global gross national income in terms of healthcare costs, reduced productivity, and increased healthcare expenditures (Walters et al., 2019). Globally, exclusive breastfeeding is regarded as the optimal form of nutrition for infants during the first six months of life (World Health Organization, 2024a).

### Contemporary empirical knowledge concerning breastfeeding

Literature has shown that breastfeeding is a predictor of positive infant health outcomes (Ihudiebube-Splendor et al., 2019; Vaivada et al., 2022). Furthermore, exclusive breastfeeding in the first six months has been shown to prevent diseases in infants such as pneumonia, new-born sepsis and diarrhoea as well as help maintain infants’ health and wellness (Vaivada et al., 2022). Breastfeeding also enhances children’s physical development as well as their neurocognitive growth (Astuti & Morgan, 2018). Additionally, breastfeeding promotes parental sensitivity and emotional attachment which has numerous psychological benefits for the mother and child (Mezzacappa & Katkin, 2002; Rivi et al., 2020). In low- and middle-income countries, breastfeeding continues to be an ideal and cost-effective source of nutrition for optimal infant growth and development. Moreover, exclusively breastfeeding a baby for the first six months of life is documented to cut mortality rates in developing countries by 13% (Fu et al., 2022).

The low prevalence rates of breastfeeding in the world can be ascribed to several factors, such as a lack of knowledge about the benefits of breastfeeding and the absence of laws supporting it in the majority of nations, particularly for working parents (Lauer et al., 2019; Pérez-Escamilla et al., 2023). Studies have shown that inadequate milk production, mother’s illness, attitudes and beliefs that their child might not enjoy human milk, cracked nipples and mothers’ return to paid employment are some of the factors that can cause exclusive breastfeeding failure (Hernández-Cordero et al., 2020). Other studies have documented negative body image concerns among mothers such as sagging breasts, abdominal size and weight gain as obstacles to exclusive breastfeeding (Acheampong et al., 2021; Rodgers et al., 2018). Literature has shown that exclusive breastfeeding rates in Sub-Saharan Africa remain low, with about 37% of infants under 6 months being exclusively breastfed (Agho et al., 2019; Otim et al., 2022). A recent study conducted in Ghana found that body image concerns and pressures negatively affect breastfeeding practices (Acheampong et al., 2021). In Zambia, researchers identified the amount of house chores and failure to be helped by spouses as barriers to exclusive breastfeeding (Tembo et al., 2015). Further, the study indicated that the challenge of breastfeeding poses a greater struggle for employed mothers (Tembo et al., 2015).

The evolving dynamics of the workforce, characterized by an increasing number of women entering the workplace, present a significant challenge to breastfeeding practices in numerous countries and cultures (Du Plessis et al., 2016). Existing literature suggests that working mothers often resort to early weaning of their infants (Bai et al., 2015; Chen et al., 2019). Nevertheless, scholarly attention on breastfeeding has predominantly centred on its health implications, creating a gap in the literature concerning breastfeeding readiness among first-time mothers. Yet literature has shown that new mothers report breastfeeding problems such as sore nipples (Kronborg et al., 2015), and concerns about having enough milk, making them vulnerable to earlier cessation, which may lead to a dread of breastfeeding a subsequent child (Palmér, 2019). Furthermore, first-time mothers report anxiety about becoming a parent, concerns about the safety of the new baby, and receiving conflicting advice (Kronborg et al., 2015). For example, a study conducted in Singapore found that first-time mothers struggled to initiate and sustain breastfeeding due to difficulty getting their babies to latch on, as well as issues dealing with contradicting advice (Jia Choo & Ryan, 2016). Research indicates that a positive breastfeeding experience is cardinal for growing confidence among first-time mothers (Hankel et al., 2019).

While global efforts have seen continuous breastfeeding advocacy in recent decades, the promotion of exclusive breastfeeding predominantly emphasizes the benefits accruing to infants, such as enhanced survival, immunity against common infections, and cognitive development. Regrettably, these campaigns often overlook the importance of assessing maternal readiness for breastfeeding and fail to incorporate the subjective perspectives of mothers regarding the potential impact of breastfeeding on their own well-being (Radwan & Sapsford, 2016). Literature emphasizes the significance of comprehending women’s perceptions and experiences of breastfeeding to offer tailored support (Radwan & Sapsford, 2016). A mother’s readiness for breastfeeding plays a pivotal role in influencing both her dedication to breastfeeding and the frequency of breastfeeding sessions for the infant (Mulyani, 2018). Enhancing infant nutrition necessitates an initial exploration of mothers’ inclination towards breastfeeding. Hence, there is a justified need for research on readiness in this context. Furthermore, a mother’s choice to breastfeed is shaped by a multitude of factors, encompassing both psychological aspects and personal perspectives (Rivi et al., 2020).

The Ministry of Health in Zambia promotes early exclusive breastfeeding, particularly during the first six months of an infant’s life, regardless of the mother’s HIV status (Spider & Mulungu, 2024). This practice is considered one of the most valuable interventions for improving child survival due to its numerous benefits, including reducing the risk of child morbidity and mortality among infants (Kumwenda et al., 2021). Interventions to promote exclusive breastfeeding among Zambian mothers have included policy reforms, formulation of guidelines mandating mothers to initiate breastfeeding within an hour of delivery (MOH, 2020), breastfeeding education campaigns, and training for healthcare providers (Spider & Mulungu, 2024). Additionally, mothers receive education about breastfeeding during both antenatal (ANC) and postnatal care (PNC) (Kamanga, 2014). Imbedded in both ANC and PNC guidelines is nutrition (Bwalya et al., 2017), health checks, information on family planning, baby care, malaria and HIV prevention (Mukonka et al., 2024). Also, recognizing that breastfeeding might be hampered by mothers’ employment, the Zambian government has increased maternity leave to 14 weeks to enhance breastfeeding of infants (Chai et al., 2018). However, despite these efforts, adherence to exclusive breastfeeding for infants from birth to six months continues to face challenges (Tembo et al., 2015). Literature has also shown that access to breastfeeding counselling services among lactating mothers remains a challenge in sub Saharan Africa (Zegeye et al., 2024).

### Rationale for the study

Although the detrimental effects of not breastfeeding on infant health outcomes are evident, there is a notable lack of research on maternal readiness for breastfeeding in Zambia. Consequently, a handful of existing studies on the topic are either outdated or engaged in elucidating the practices and factors that influence exclusive breastfeeding in general. Given this backdrop, there is a pressing need to explore and comprehend the perspectives of first-time mothers, specifically regarding their readiness to breastfeed. Acknowledging the existing gaps, this study attempts to contribute to bridging the gaps by elucidating the lived experiences of first-time mothers with readiness to exclusively breastfeed and identify the faced challenges. It is anticipated that this study could offer valuable insights into how public health interventions can help first-time mothers prepare for the breastfeeding experience.

### Aim

This study aimed to explore exclusive breastfeeding readiness and experiences of first-time mothers in Zambia.

## Methods

### Study design

The present study adopted a qualitative exploratory methodology (Biggerstaff & Thompson, 2008), using an interpretative phenomenological approach (Frechette et al., 2020) to delve into the phenomenon of exclusive breastfeeding readiness as experienced by first-time mothers. Previous literature has consistently highlighted the suitability of phenomenological designs for studying lived experiences. Langdridge (2007) and Alase (2017) emphasize that phenomenological approaches focus on the experiences as perceived by the informants, making them ideal for capturing the subjective, personal aspects of phenomena like exclusive breastfeeding readiness. Given the complex and highly personal nature of breastfeeding, the interpretative phenomenological approach was deemed to be particularly well-suited for this study as it allowed us to collect rich and detailed narratives, to explore the subjective experiences and personal aspects of breastfeeding; thus, offering a contextual understanding of the lived experiences of first-time mothers, which can inform healthcare practices and support systems aimed at promoting exclusive breastfeeding (Frechette et al., 2020).

### Study setting

The research was conducted in Lusaka, a city characterized by a substantial population estimated at 3,079,964 people (Zambia Statistical Agency, 2022). Data from the Zambia Demographic Health Survey indicates that 70.0% of infants in urban areas like Lusaka receive exclusive breastfeeding, compared to national rates ranging from 64.9% to 65.2% (Ministry of Health, 2024). Nevertheless, no prevalence data exists on exclusive breastfeeding among first-time mothers, even in light of these statistics. Lusaka District has an estimated 735,361 women of childbearing age (Hibusu et al., 2024), most of whom work in the informal sector. The demanding nature of work in both informal and formal sectors among mothers in Lusaka may hinder their ability to exclusively breastfeed. Therefore, the urban environment of Lusaka was deemed suitable for this research. The three health facilities, from which mothers were selected, comprised two governmental facilities and one private facility. All selected hospitals provided prenatal, childbirth, and postnatal care services which includes breastfeeding teaching and support to mothers.

### Sample size and sampling

Given the proposed study design, purposive sampling (Luborsky & Rubinstein, 1995) was implemented to acquire the participation of respondents, which is a widely used sampling technique in conjunction with qualitative research. In this regard, this sampling technique was used to identify and select respondents that had knowledge and experience (Creswell & Clark, 2017) in terms of breastfeeding i.e., equivalent to what is referred to as identification and selection of information-rich cases (Patton, 2002).

With regards to sample size, the acquisition of a comprehensive understanding of the phenomenon in question was carried out by continuation of sampling until no new substantive information was unfolding, i.e., until saturation was deemed upheld (Miles & Huberman, 1994; Sandelowski, 1995).

### Participants

The study participants consisted of 17 first-time mothers recruited purposefully from the waiting area of the three participating hospitals’ postnatal outpatient departments. Study participant number of 17 mothers was the point at which data saturation was achieved. Purposive sampling helped to select mothers of different socioeconomic status and education. All the participants identified themselves as mothers who had given birth to their children. The inclusion criteria were as follows: participating women should be currently living in Lusaka and receiving maternal care from one of the selected hospitals; being first-time mothers; and having given birth to the currently breastfed child. Furthermore, the mother must have been breastfeeding the baby exclusively from birth to a minimum of six months at the time of recruitment. The exclusion criteria were participants who were not first-time mothers and mothers who could not breastfeed their babies for medical reasons. The rationale underlying the inclusion and exclusion criteria was based upon the previous empirical studies including the relevance of sex and not gender identities in conjunction with the act of breastfeeding (Gribble et al., 2022).

### Recruitment and selection of participants

Mothers attending any of the selected healthcare centres for routine visits to the paediatric outpatient department were provided with information letters (containing an invitation to participate, the purpose of the study, what their participation would entail, and the procedures of the study) while waiting. The selection process of the mothers involved the use of purposive sampling, supplemented by snowball. Once eligible first-time mothers were identified and agreed to participate, mothers were asked to sign a written consent form, and a mutually convenient time and location for the interview was arranged based on the mothers’ preferences. The initial identified mothers helped to recruit other mothers. In-depth individual interviews were conducted to collect data, which allowed the researcher to elicit mothers’ views on readiness and experiences. The interview guide was created in line with the study objectives and included open-ended questions on how the mothers prepared themselves for breastfeeding, how they experienced exclusive breastfeeding, and what they considered the most important resources needed to breastfeed exclusively. The interview guide was pilot-tested on a sample of mothers who did not meet the selection criteria for participation in the main study. Following the pilot test, no amendments to the guide were deemed necessary.

All interviews were audio-recorded using a recorder with the consent of participants and transferred onto a password-protected computer for the secure protection of information and participants’ anonymity. The mean time for the interviews was 30 minutes, ranging between 28 and 45 minutes.

### Data analysis

The data was analysed manually after a verbatim transcription of the interviews. The transcripts were read and reread by the authors to familiarize themselves with the data and fully understand the mothers’ breastfeeding experiences. The analysis started with the authors reading all transcribed interviews several times to familiarize themselves with the data. The transcripts were later read systematically, manually highlighting sentences with meaning units, and identifying initial codes, after which meaning units were extracted. Further, all the meaning units were later condensed, and sub-categories abstracted and labelled. The identified sub-categories were later grouped and compared leading to the development of categories and subsequently, thematic description was constructed. The identified theme was later cross-checked by all the researchers to ensure consensus and to increase trustworthiness. Research literature has demonstrated that a researcher’s positionality is inherently intertwined with the research process (Palaganas et al., 2017). Recognizing this, the researchers in this study adopted a reflexive approach throughout the data collection, analysis, and writing processes. They continuously reflected on their subjectivity, particularly their values, professional experiences, and assumptions, to mitigate potential biases and enhance the credibility of the data and findings (Darawsheh, 2014).

### Trustworthiness

Trustworthiness of the findings was ensured by adhering to Guba’s four key trustworthiness criteria components: credibility, transferability, dependability, and conformability (Guba, 1981).Credibility was enhanced through reflexive practices and by eliciting rich, detailed descriptions of participants’ exclusive breastfeeding experiences. To facilitate potential replication, an in-depth description of the methods and findings was provided. To enhance the dependability of the findings, the research team employed a rigorous data analysis process. This process involved independent coding and theme identification by multiple team members, followed by consensus-building discussions to confirm the final theme.

### Ethical considerations

This study was conducted following the Helsinki Declaration on ethical principles involving human subjects (General Assembly of the World Medical Association, 2014). Ethical approval was sought and granted by the University of Zambia, School of Humanities and Social Sciences Research Ethics Committee approval HSSREC-2023-AUG-033.

The enrolled mothers were briefed on the study and given ample time to review and inquire before making a decision on their participation. The information letter elucidated the study’s objectives, the participants’ voluntary involvement, and their right to withdraw at any point without justification or repercussions, and an assurance of confidentiality regarding their information. Subsequently, they were asked to provide written informed consent and participants were also required to consent for interviews to be recorded before the commencement of the interview. The confidentiality and anonymity of the participants were ensured by not retaining any identifying information and the interviews were given unique codes of identification. These codes were secured, together with the interview recordings, in a password-protected computer accessible only by the principal investigator, while the transcripts were available to the research team for triangulation.

## Results

In terms of maternal characteristics, the participating mothers were between 19 and 35 years old with a mean age of 27 years, while their babies ranged in age from 4 to 24 months. All the participants reported having attended antenatal care (ANC) clinics during pregnancy and adhering to exclusive breastfeeding practices. The analysis yielded one main theme Breastfeeding Readiness—A Multifaceted Approach with four categories: Exclusive Breastfeeding Readiness and Motivation, Support Networks in the Breastfeeding Journey, Navigating the Exclusive Breastfeeding Journey, and Perceived Resources to Support Exclusive Breastfeeding Readiness. See Table 1 in Appendix.

### Breastfeeding readiness—a multifaceted approach

The mothers’ experiences highlighted the complex and multifaceted nature of breastfeeding readiness that encompasses various aspects of a mother’s preparation for exclusive breastfeeding. It involves personal motivation, support systems, experiences, and access to resources. This comprehensive approach recognizes that successful breastfeeding depends not only on individual readiness but also on the broader context of family, community, and healthcare support. Mothers reflected experiences addressing these interconnected factors, and meant that they can be better equipped to navigate the challenges and joys of the exclusive breastfeeding journey.

### Exclusive breastfeeding readiness and motivation

This category was built on two sub-categories; preparedness, and motivation to exclusively breastfeed. The sub-categories preparedness to exclusively breastfeed revealed ideas around knowledge preparedness, practical preparedness, and mental preparedness. Regarding knowledge preparedness, the majority of the mothers expressed being well-informed and had enough breastfeeding information (i.e., benefits of breast milk, process of feeding), despite a small number indicating feeling unprepared and lacking sufficient knowledge. Mothers cited various sources for their knowledge which included the internet, interactions with significant others, personal research, healthcare providers, and reading materials. One participant reported:

I did a lot of research concerning breastfeeding and I read a lot about new mothers who experience maybe low production of milk so I was interested, I had to go on Google and research to find out okay so what causes may be low production of milk, how to prevent that from happening or how to help prevent that from happening. (Interviewee 1)

Similar to knowledge readiness, the practical preparedness also yielded varying perspectives among mothers, with some mothers indicating readiness, while others expressed unpreparedness. An example of practical preparedness was the purchase of breast pumps to ensure sufficient breast milk production, and learning how to latch the baby. As one mother put it:

I bought a breast pump. What I heard is that the more you pump, or the more the baby is put on the breast the milk tends to come out, so I made sure I got a breast pump. (Interviewee 6)

However, the majority of the mothers disclosed being unprepared for the practical aspects of breastfeeding such as positioning the baby, a comfortable position for the mother, and proper ergonomics. As one mother conveyed:

I was getting some ideas from my mum but practicality, I was not prepared at all. (Interviewee 4)

The mothers’ perspectives on their mental readiness for breastfeeding are related to the sub-category of mental preparedness. Similarly, opinions on mental preparedness varied, with many mothers expressing a lack of mental readiness while a few acknowledged feeling mentally unprepared. Nearly all mothers initially believed that breastfeeding was instinctual for mothers due to childbirth, leading to a sense of being mentally unprepared for the responsibilities and challenges it entails. For example, one mother said:

Mentally I didn’t prepare for anything, I just thought it’ll be automatic, to be honest. I thought that I’ll give birth, I’ll just put the baby on the boob and the baby would start sucking. (Interviewee 8)

Mothers who expressed mental readiness emphasized the importance of preparing mentally in a similar manner to how they prepare materially for the arrival of the baby. For example, a mother shared:

I started preparing myself mentally and physically because I knew to say the time I’ll give birth to this child, it’ll need to be fed. So the same way that you prepare for baby clothes and baby that’s how I prepared mentally myself as well. (Interviewee 7)

The category “*exclusive breastfeeding readiness and motivation” also brought out* motivating factors that drove first-time mothers to embrace breastfeeding. Reasons advanced by mothers revolved around self-motivation, and external influences such as familial and hospital influences. Regarding self-motivation, mothers narrated about their beliefs and perceptions of the nutritional benefits of breastfeeding for the infant’s health. One participant expressed this sentiment:

I chose to breastfeed because I feel breast milk is the healthiest food you can give to a newborn baby. I consider breast milk to be the healthiest for the child and for the fact that they get the nutrients directly from the mother. (Interviewee 7)

Another mother articulated: “*I think health-wise also I felt like it was important for me to breastfeed for 90 days at least exclusively. I needed to try as much as it was very difficult to breastfeed.”* (Interviewee 8)

Mothers also talked about their inner motivations being cardinal in embracing exclusive breastfeeding, driven by the belief in the superior health benefits of breast milk. As described by one mother:

For me, it was mainly the benefits that I had read about, during the antenatal visits as well there is this chart that they show you, … they say breastfeeding exclusively is important for the baby because of all the nutrients. (Interviewee 6)

The hospital and the family were described as having a significant influence on mothers’ decisions to consider exclusive breastfeeding, as evidenced by the sub-category of external influence. Mothers mentioned being inspired by their maternal figures, including mothers, grandmothers, aunties, as well as guidance received from healthcare facilities regarding the importance of exclusive breastfeeding. Some mothers talked about feelings of being pressured, particularly by their own mothers who consistently reminded them about the significance of breastfeeding. One mother shared,

I would say there was a little bit of pressure from my mum who was staying with us at the time because she and her sister who’s a nurse, would always call to check if I was breastfeeding the child. (Interviewee 16)

Additionally, mothers highlighted how guidance from the hospital staff served as a motivating factor in their decision to pursue exclusive breastfeeding, with one mother stating,

The hospital I gave birth from, told me breastfeeding is important. (Interviewee 14)

### Support networks in the breastfeeding journey

This category revealed the spectrum of support as a key factor in helping mothers to exclusively breastfeed. Each mother interviewed indicated receiving various forms of assistance from friends, family, and the community. In terms of breastfeeding support, the majority of mothers mentioned consistently receiving counselling, information, nutritional guidelines, and aid with expressing breast milk. Additionally, mothers highlighted the importance of receiving social-emotional support during challenging moments in their breastfeeding journey, providing crucial encouragement when they felt overwhelmed or contemplated giving up. As one mother shared,

My mother always calls to ask are you breastfeeding? Are you eating correctly? How’s your diet? Also, my aunt calling to ask or advise if your breasts are sore, this is what you do – put a peal of cabbage in the fridge and put it over the breast. (Interviewee 13)

Furthermore, mothers emphasized the supportive role of friends, with one mother expressing gratitude for friends who shared their own experiences and reassured her that she was not alone in facing challenges such as balancing breastfeeding with work commitments. Community support was also highlighted as a vital component of their breastfeeding journey, with mothers citing instances of moral backing and encouragement from community members who exhibited non-judgemental attitudes towards breastfeeding. As articulated by one mother,

The community in general is supportive, if you have to feed your baby whether you are in a public place, even on public transport like a bus, no one will ever make you feel uncomfortable for it …. Nobody judges you for feeding your child at any place, and any time. That helps. (Interviewee 17)

### Navigating the exclusive breastfeeding journey

The category of “*Navigating the Exclusive Breastfeeding Journey*” sheds light on the diverse experiences of first-time mothers during the breastfeeding process, encompassing both positive and negative aspects. The category was developed around two sub-categories: favourable and unfavourable experiences.

Within the sub-category of favourable experiences, the narratives highlighted the bonding opportunities and sense of fulfilment that breastfeeding fosters. Mothers who successfully breastfed shared positive encounters, underscoring how exclusive breastfeeding facilitated bonding with their children. For instance, one mother stated,

It is fulfilling because first of all, you are providing the nutrition for your baby. But I also think in those moments even when you are tired, and this baby is on the breast. It’s the way the baby looks at you and holds your hand, your fingers … ultimately it’s knowing that you are keeping the baby alive that makes it worthwhile. (Interviewee 6)

This quote illustrates how breastfeeding gave mothers a sense of fulfilment and strengthened their bond with the baby. Another mother expressed her belief that successful breastfeeding was pivotal for personal achievement, stating, “*I think it’s a good feeling aside from connecting with the baby*” (Interviewee 9), emphasizing the significance of breastfeeding in her sense of accomplishment.

In addition to favourable experiences, the findings also revealed unfavourable experiences encountered while navigating exclusive breastfeeding. Mothers emphasized how challenging it was to manage exclusive breastfeeding. A prevalent difficulty reported by many mothers was the management of breast soreness, pain, mental stress, physical changes, and exhaustion. Further, they described breastfeeding as an emotionally demanding journey that left them physically and mentally drained. The majority of the participating mothers expressed experiencing mental and emotional distress due to breastfeeding. As one mother reflected,

It was mentally draining. I was trying to be that mum that gives my baby the breastmilk, but it was very hard for me because my milk wasn’t coming out, even if it did it was about 20mls and he wasn’t getting full and he was failing to latch on the other boob. So, it became really hard because the one he wasn’t able to latch on at least seemed to have more milk. (Interviewee 12)

The presence of breast sores was a common feature in the experiences of mothers. These challenges predominantly stemmed from physical sores and pain associated with breastfeeding. Mothers frequently reported enduring painful nipples, which posed difficulties in exclusively breastfeeding their infants. One mother expressed,

Breast sores were the worst experience ever; it was quite painful. It was very painful and then you are told to continue putting the baby on the breast. (Interviewee 2)

Another mother shared, “*When the baby is starting to suck, if you are not used your nipples will develop sores. Whereby they’ll be like blisters. Because of that, there will be pain and it will be very much discomforting*.” (Interviewee 11)

Additionally, mothers mentioned experiencing irritation and discomfort during breastfeeding, with one mother expressing, “*Because like when the child is sucking I was feeling like maybe the baby was sucking the person out of me*.” (Interviewee 1)

Difficulty in latching the baby onto the breast was identified as a source of anxiety among mothers. Those who encountered challenges with proper latching during breastfeeding expressed feelings of inadequacy, frustration, and overwhelmed thoughts of failure. This sentiment is exemplified by a mother who shared,

I struggled with how to hold the baby when breastfeeding, the baby was not able to latch properly, and my milk production was very low so I couldn’t feed the baby properly. This made me very anxious, and it just made the experience very horrible for me. (Interviewee 15)

These emotional challenges were observed to affect first-time mothers in various ways, with their predominant struggles revolving around latching challenges, insufficient milk production, and managing emotional distress and concerns. Closely linked to these emotional struggles were reports of low milk supply. First-time mothers described the challenges they faced due to limited milk production, which compelled some to supplement breastfeeding with formula. Many mothers expressed a shared sense of dissatisfaction and disappointment with their exclusive breastfeeding journey attributed to inadequate milk production. One mother explained,

I had challenges in terms of milk production, so I would have to drink Chibwantu (local maize meal drink), I would have to take black tea with lemon and just try all sorts of things for me to just increase the milk production. (Interviewee 14)

Physical changes to their bodies represented another facet of the mothers’ breastfeeding journey. Mothers acknowledged that breastfeeding had led to alterations in their bodies, with some expressing feelings of detachment from their physical selves. They discussed the differences in the appearance of their bodies and breasts, reporting that it was often challenging and emotionally distressing. For example, one mother remarked,

Nobody explains to you that your breasts might become three times your regular size and you go three bra sizes up, these are all things that you’re experiencing but no one mentions them. (Interviewee 6)

Feelings of increased hunger were closely associated with bodily changes. Mothers described how breastfeeding drained their energy and altered their eating patterns due to increased appetite. Each mother noted a surge in appetite and intense hunger, a sensation they had not experienced before. As expressed by one mother,

For me my breastfeeding experience … I think what took me by surprise firstly were the changes that happen in your own body when you are breastfeeding. Nobody told me about the hunger, the type of hunger that comes with breastfeeding. It was confusing at first because I was like I ate breakfast … the hunger like you haven’t eaten for 4 days. (Interviewee 7)

Mothers who opted for exclusive breastfeeding noted that this choice influenced various aspects of their personal lives. The disruption encompassed changes in their physical appearance and a lack of personal time for self-care.

### Perceived resources to support exclusive breastfeeding readiness

The category of *perceived resources to support exclusive breastfeeding readiness* was supported by four sub-categories: Information needs, dietary considerations, mental readiness, and improved maternity healthcare services.

In terms of perceived informational needs, mothers expressed that their breastfeeding journey commenced with limited information regarding the challenges and nuances of breastfeeding. They highlighted key issues such as breast sores, difficulties in latching the baby, and managing babies who were teething as novel experiences. Mothers believed that these topics should have been addressed during prenatal care, yet they were not covered in the existing prenatal programmes. One mother articulated,

I feel like we often overlook this thing of breastfeeding, I feel like right when you are pregnant, it’s important to maybe get beforehand information so that you are aware; that this is what could happen if you deliver. (Interviewee 11)

In addition to acquiring adequate information on breastfeeding, the importance of mental preparedness was underscored. Mothers emphasized the significance of maintaining a positive mental attitude towards breastfeeding, meaning that it greatly aided exclusive breastfeeding. First-time mothers expressed feeling inadequately prepared mentally for the breastfeeding journey. Some mothers suggested that being mentally prepared and informed about potential mental health challenges during the breastfeeding journey could enhance their readiness for exclusive breastfeeding. As highlighted by one mother,

It’s just to prepare your mind and then you just need to be positive because once you have a positive mind, everything else flows, even breastfeeding. (Interviewee 10)

Moreover, another sub-category that emerged was improved maternal healthcare services. It was evident that the existing maternal healthcare services provided insufficient preparation for first-time mothers. Mothers expressed dissatisfaction with the current system, which offers prenatal care services in a group setting without considering the unique needs of first-time mothers. They highlighted that crucial issues common to their experiences such as latching difficulties, breast sores, mental health challenges, and other breastfeeding concerns, were often overlooked. As one mother emphasized,

Maternity talks with first-time mothers should be separate from others and should be at least twice a month. (Interviewee 3)

Another mother remarked, “*antenatal services should provide sufficient information … you sit as mothers, they’ll give you information about family planning, not breastfeeding.”* (Interviewee 16)

Some mothers highlighted the lack of essential topics in current maternal health services. One mother shared her experience of learning proper latching techniques from online sources like YouTube rather than from hospital resources. Mothers also identified certain deficiencies in the existing maternal healthcare services. As one mother pointed out, the emotional aspects of exclusive breastfeeding, including fatigue, stress, anxiety, and sadness, were often neglected in maternal healthcare discussions despite their significant impact on milk production and breastfeeding success.

The sub-category of dietary considerations was closely linked to maternal healthcare services. Mothers emphasized the importance of addressing nutritional needs and dietary adjustments specifically during prenatal care services. Common experiences reported included a lack of awareness regarding changes in nutritional requirements during breastfeeding and constant feelings of hunger. Mothers noted that dietary modifications were essential for breastfeeding, yet this aspect was not addressed during prenatal appointments. The majority of the participants indicated that they acquired this knowledge from sources outside the hospital. For example, one mother mentioned,

I learnt from my parents … food or drinks for me to take to have enough milk for the child … Tonga traditional drink, Chibwantu and tea. (Interviewee 1)

The quotes revealed a deficiency in breastfeeding knowledge among first-time mothers. The lack of information about breastfeeding and achieving a proper latch was reported by most mothers.

## Discussion

Breast milk possesses unique qualities that position breastfeeding as the optimal choice for a baby’s primary source of nourishment (Lassi et al., 2020). Despite the well-documented health advantages of breastfeeding, there is a scarcity of research on exclusive breastfeeding preparation and experiences among first-time mothers. The current study aimed to explore the readiness for and experiences of exclusive breastfeeding among first-time mothers in Zambia. Aligning with this objective, the study revealed diverse views on readiness, with most first-time mothers indicating knowledge readiness, while a small number expressed mental and practical readiness. The experiences of mothers in navigating exclusive breastfeeding journeys were characterized by a mix of favourable and unfavourable encounters. Furthermore, the study identified perceived resources such as informational needs, dietary considerations, mental readiness, and improved maternity healthcare services that could facilitate the exclusive breastfeeding journey for first-time mothers. Given the limited research on breastfeeding readiness and experiences among first-time mothers in the Zambian context, this study holds significant importance as it contributes valuable insight to the existing literature.

Research indicates that women who engage in early preparation for breastfeeding are likely to be more physically and psychologically prepared for exclusive breastfeeding (Mulyani, 2018). Regarding first-time mothers’ readiness to breastfeed, our study revealed that most mothers possess knowledge about breastfeeding, but were lacking in both mental and practical preparedness for the demands and experiences that came with exclusive breastfeeding. The internet, hospitals, friends, grandmothers, aunties, and mothers played as valuable sources of knowledge for the mothers in our study. These findings clearly demonstrate the diverse sources that contribute to mothers’ acquisition of breastfeeding knowledge. Our results align with other studies within Zambia and in the United States of America that highlight the influence of friends, grandmothers, aunties, mothers, and the internet on mothers’ breastfeeding knowledge (Kaluba et al., 2023; Kornides & Kitsantas, 2013; Mwiza et al., 2023; Nchimunya et al., 2015). The reason for similarities between our study and the ones mentioned above is that participants had great knowledge about breastfeeding as well as strong support systems, which helped with readiness to breastfeed as well as sustenance of the practice.

Interestingly, our findings also highlight a knowledge-behaviour gap among mothers. Despite the majority of mothers in our study acknowledging that they were knowledgeable about breastfeeding, they also admitted to being mentally and practically unprepared for the actual act of breastfeeding. This disparity emphasizes that having knowledge does not necessarily translate into readiness on a practical and mental level. These findings align with previous studies that have identified a lack of breastfeeding preparedness among mothers (George, 2005). Given the breastfeeding challenges narrated by mothers in the current study, it is evident that many mothers were unprepared mentally and practically. The findings underscore the deficient role that maternal healthcare services play in the relay of proper knowledge and aid to first-time mothers, necessitating room for improvement.

The results of our study revealed varying experiences among mothers regarding breastfeeding. While some mothers talked about their positive experiences, such as forming connections and bonding with their children, others shared negative experiences, including issues like breast sores, pain, stress, latching difficulties, low milk production, and mental challenges. Our study indicates that mothers encountered multiple breastfeeding challenges related to physical (breast sores, sore nipples), practical (low supply of milk), and mental aspects. The findings resonate with existing research that has documented similar issues related to mental challenges (Giannì et al., 2020), and practical obstacles (Mahurin-Smith, 2023; Mitchell & Johnson, 2022). The experiences of mothers in our study were characterized by feelings of inadequacy due to the physical and mental demands related to breastfeeding. Most mothers expressed uncertainty about how to address challenges concerning breast sores, pain, and insufficient milk supply. Furthermore, the physical and mental toll of exclusive breastfeeding underscores the necessity for tailored mental and practical support for new mothers.

While our study findings align with existing research on the lived experiences of mothers in various aspects of breastfeeding, there were significant variations in results. Our findings align with the existing research by highlighting the crucial role of social support from mothers, grandmothers, and other influential people in assisting breastfeeding mothers in navigating the challenges they face. This research reinforces the well-known African proverb that emphasizes the collective effort required to raise a child (it takes a village to raise a child) (Yendork & Somhlaba, 2015). Notwithstanding the significance of social relationships, it is essential to recognize that advice provided to first-time mothers may not always be medically accurate. However, due to their lack of experience, it can be challenging for mothers to discern this. With regards to the variations in our findings, they are exhibited in the relay of information conveying that antenatal care services often overlook many of the difficulties faced by first-time breastfeeding mothers. There is a significant likelihood that mothers may receive misinformation or advice unsupported by medical evidence to guide them through the breastfeeding process (Lubbe et al., 2022). In contrast to existing research, our study highlights the lack of implemented person-centred care, procured through a lack of involvement of first-time mothers in the care services provided. Each first-time mother is an individual with distinct abilities and needs from other first-time mothers. The qualities and needs distinguish every first-time mother, and our findings suggest that these distinguishable qualities tend to be missed or neglected by healthcare professionals in antenatal care services (Massare et al., 2023). Our results suggest that healthcare providers and antenatal healthcare services should prioritize providing comprehensive prenatal and postnatal care focused on breastfeeding for first-time mothers. The literature emphasizes the importance of providing optimal breastfeeding support for the health of both mothers and their infants (James et al., 2020). Additionally, healthcare professionals should consider each mother’s unique circumstances when providing breastfeeding guidance (Hvatum & Glavin, 2017). As was reported, the current group-based antenatal services, conducted for both first-time and experienced mothers in one group failed to acknowledge that first-time mothers’ needs differ thus, this “one shoe fits all” kind of approach to antenatal services needs to be reconsidered. Furthermore, maternal healthcare providers should be attentive to the needs of first-time mothers, who might require additional support as they are novices with no prior knowledge and may not be mentally and practically prepared for the breastfeeding experience.

### Methodological limitations

Despite these results, our study has a few limitations. Since the study used a qualitative approach, it is not possible to transfer the findings to all first-time mothers in Zambia. Additionally, an important aspect of breastfeeding, as defined by the World Health Organization (WHO), is that it encompasses feeding an infant either directly at the breast or with expressed human milk (WHO, 2024a). However, this aspect was not discussed with the participating mothers in our study, which could have provided valuable insights into the practice of exclusive breastfeeding. We recommend that future studies investigate this dimension to enhance our understanding of current breastfeeding practices and the readiness of first-time mothers to provide exclusive breastfeeding. A limitation of this study is its reliance on maternal recall to explore experiences of exclusive breastfeeding. Given that the children’s ages ranged from 4 to 24 months, there is a possibility of recall bias, particularly among mothers of older children. This potential bias arises because exclusive breastfeeding typically occurs within the first 6 months of an infant’s life, and some participants were recalling events from up to 18 months prior.

However, despite these limitations, to ensure the trustworthiness of the findings, the study employed Guba’s four key trustworthiness criteria components: credibility, transferability, dependability, and conformability (Guba, 1981). Given the scarcity of studies on breastfeeding readiness in Sub-Saharan Africa, particularly in Zambia, this study contributes valuable insights to the existing body of knowledge on the exclusive breastfeeding experiences of first-time mothers.

Another possible perceived limitation worth pointing out is the use of sexed based terms in our manuscript and a sex-based study with regards to the use of the words “women” and “mothers”. In this regard, the emphasis in our study was on the sex of the respondents and not on gender identities, with the inclusion criteria set at female, with the rationale being based upon previous research that has included the relevance of sex and not gender identities in conjunction to the act of breastfeeding. Additionally, the rationale was not to be discriminatory but to take into account what is relevant in terms of women’s needs in conjunction with the act of breastfeeding. While it is important to implement a novel approach when considering sex and gender, rendering a more correct, sensitive, and inclusive data collection, it is concurrently as important to consider the ramifications of the desexing language of female reproduction as has been conveyed by Gribble et al. (2022). The purpose of our study has been to highlight the unique experience and needs among women as first-time mothers in Zambia regarding breastfeeding, which according to Gribble et al. (2022) is not shared by other groups. It has been expressed that utilizing accurate language and definitions when relaying and setting up research with regard to female reproduction and newborn care is vital and necessary because not all groups have the same health needs or experiences as first-time mothers. Hence there is a need to distinguish between groups (Gribble et al., 2022). In the words of Gribble et al. (2022, p. 7), “When sex is meant, refer to ‘sex’”. With regards to our study, sex was meant and thus, we refer to “sex” when using the terms “women” and “mothers”.

Thus, desexing the language of female reproduction was not seen as relevant within the scope of our study, to be inclusive of women, not undermining their lived experience with the act of breastfeeding by avoiding the term mother in its sexed sense. Thus, we choose to include terminology that does not render a lack of clarity on who is being alluded to, as well as risk obstructing the challenges faced by first-time mothers in relation to breastfeeding because they are female, as well as obstruct the supply of care and corrode the needs of mothers and their infants.

### Significant implications of findings

In light of our findings, we recommend maternal healthcare providers dedicate efforts to preparing mothers both mentally and practically for the breastfeeding journey. For example, sometimes breast milk will not come immediately and a little more effort such as specific nutrition should be followed to boost it and to teach mothers how to position and latch the baby on the breast (Pados & Camp, 2024). As highlighted by one scholar, women who engage in early preparation for breastfeeding are more likely to be physically and psychologically prepared for exclusive breastfeeding (Mulyani, 2018). Secondly, we recommend that antenatal care programmes be specially tailored for first-time mothers, focusing on addressing the challenges encountered during the breastfeeding journey. It is important to recognize that each first-time mother is a unique individual with distinct abilities, specific challenges, and varying needs. Our findings underscore the importance of promoting person-centred maternity care (PCMC) tailored to the individual needs of first-time mothers, providing them with mental and practical support for an exclusive breastfeeding experience before, during, and after childbirth (Sudhinaraset et al., 2017). This approach aligns with the WHO’s standards for improving the quality of maternal and antenatal care (World Health Organisation, 2016), particularly reflecting standard 6. Standard 6 emphasizes the importance of providing tailored emotional support that is responsive to the diverse needs of women and empowers them in their roles as mothers. To achieve this, it is essential to view first-time mothers as active partners in the care provision process. This collaborative approach requires a harmonious balance between the goals, expertise, and resources of first-time mothers and healthcare professionals, fostering a collaborative partnership.

However, given the scarcity of studies on breastfeeding readiness in Sub-Saharan Africa, particularly in Zambia, this study contributes valuable insights to the existing body of literature on the exclusive breastfeeding experiences of first-time mothers. Policymakers can utilize these findings to develop targeted interventions that focus on providing comprehensive prenatal and postnatal care, specifically tailored to support first-time mothers in their breastfeeding journey in the form of PCMC. Nursing and midwifery practices could benefit from incorporating person-centred strategies to address the mental and practical challenges faced by new mothers. Implementing PCMC ensures that they receive the necessary support and guidance, potentially enhancing a deeper understanding of first-time mothers’ experiences and facilitating improved communication between these mothers and healthcare professionals (Sudhinaraset et al., 2017). In terms of education, these results emphasize the importance of enhancing breastfeeding education programmes to equip mothers with the knowledge and skills needed to navigate the complexities of exclusive breastfeeding successfully. Furthermore, the research underscores the need for further studies in this area to expand the existing literature and provide more insights into breastfeeding readiness, experiences, and challenges faced by first-time mothers in Sub-Saharan Africa, including Zambia. Moreover, subsequent studies could explore how socioeconomic factors might influence breastfeeding readiness experiences in first-time mothers.

## Conclusions

Although first-time mothers have knowledge of exclusive breastfeeding, mentally and practically, they are not ready to do so successfully. The findings of the current study underscore several difficulties experienced by first-time mothers during their exclusive breastfeeding journey. These results highlight the need for improved antenatal care provided to new mothers, with a focus on an enhanced provision of practical breastfeeding education, instruction, and coping strategies for example latching babies, nutritional support with focus on milk production, and how to manage breast sores.

## Data Availability

The datasets generated and/or analysed during the current study are not publicly available due [restrictions by the Research and Ethics Committee at the University of Zambia to protect the participants’ privacy] but are available from the Principal Investigator on reasonable request. The data used is stored at www.unza,zm.

## Appendix

**Table AI.** An overview of the overarching theme, categories and sub-categories reflecting first-time mothers’ readiness experiences to exclusively breastfeed their babies.

Theme	Breastfeeding Readiness—A Multifaceted Approach			
Categories	Exclusive Breastfeeding Readiness and Motivation	Support Networks in the Breastfeeding Journey	Navigating the Exclusive Breastfeeding Journey	Perceived Resources to Support Exclusive Breastfeeding Readiness
Sub-categories	Preparedness to exclusively breastfeed	Immediate family	Favorable experiences	Information needs
	Motivation to exclusively breastfeed	Community support	Unfavorable experiences	Diet
				Mental readiness
				Enhanced maternity healthcare services
